# High expression of MMP19 is associated with poor prognosis in patients with colorectal cancer

**DOI:** 10.1186/s12885-019-5673-6

**Published:** 2019-05-14

**Authors:** Zaiping Chen, Guiyang Wu, Fubo Ye, Guoping Chen, Qinghao Fan, Hao Dong, Xiongwen Zhu, Chongshan Wu

**Affiliations:** Department of General Surgery, Taizhou Municipal Hospital, Medical College of Taizhou University, Taizhou, 318000 Zhejiang Province China

**Keywords:** Colorectal cancer, MMP19, Survival analysis

## Abstract

**Background:**

Matrix metalloproteinase 19 (MMP19) is a member of zinc-dependent endopeptidases, which have been involved in various physiological and pathological processes. Its expression has been demonstrated in some types of cancers, but the clinical significance of MMP19 in colorectal cancer (CRC) has not been reported. Thus, we aimed to analyze the clinical significance of MMP19 in CRC in present study.

**Methods:**

The expression of MMP19 was first explored in The Cancer Genome Atlas (TCGA) cohort, and then validated in the GSE39582 cohort and our own database. Clinicopathological features and survival rate were also investigated.

**Results:**

MMP19 was found to be a predictor for overall survival (OS) in both univariate (hazard ratio [HR]: 1.449, 95% confidence interval [CI]: 1.108–1.893, *P* = 0.007) and multivariate survival analyses (HR: 1.401, 95% CI: 1.036–1.894, *P* = 0.028) in the TCGA database. MMP19 was further validated as an independent factor for recurrence free survival in the GSE39582 database by both univariate analysis (HR: 2.061, 95%CI: 1.454–2.921, *P* < 0.001) and multivariate analysis (HR = 1.470, 95% CI: 1.025–2.215, *P = 0.032*). In an in-house cohort, MMP19 was significantly upregulated in CRC tissues when compared with their adjacent normal controls (*P* < 0.001). Ectopic MMP19 expression was positively associated with lymph node metastases (*P* = 0.029), intramural vascular invasion (*P* = 0.015) and serum carcinoembryonic antigen levels (*P* = 0.045). High MMP19 expression correlated with a shorter OS (HR = 5.595; 95% CI: 2.573–12.164; *P* < 0.001) and disease free survival (HR = 4.699; 95% CI: 2.461–8.974; *P* < 0.001) in multivariate cox regression analysis.

**Conclusions:**

Expression of MMP19 was upregulated in CRC. High expression of MMP19 was determined to be an independent and poor prognostic factor in CRC. These results suggest that MMP19 may be a good biomarker for CRC.

## Background

Colorectal cancer (CRC) is the third most common and the third leading cause of cancer-related death in the United States [1]. In China, both the incidence and mortality rate of CRC has been increasing, and CRC is ranked as the third leading cause of cancer-related deaths [2]. The development of distant metastasis is the main reason of cancer-related death regardless of effective surgical procedures and systemic chemotherapy. Approximately 20–25% of patients are initially diagnosed with synchronous metastases, of which approximately 50% ultimately develop metachronous disease after colectomy [3, 4]. The survival outcome of CRC is mainly determined by tumor stage and some other clinicopathological factors. However, the heterogeneity of this disease makes it difficult to predict patient prognosis with these traditional factors [5]. Recent genetic and molecular analyses of CRC have identified a set of predictive biomarkers, including RAS status, BRAF mutation and mismatch repair protein expression, that can aid in the identification of patients who are at high risk of disease progression or recurrence [3, 6–8]. Although available biomarkers are commonly used for predicting long-term outcome, some previous studies have reported that a proportion of patients are misdiagnosed [7, 8]. Therefore, the identification of novel markers that can be used to screen various prognostic risk subgroups to guide individual treatment is urgently needed.

Matrix metalloproteinases (MMPs) are zinc-dependent endopeptidases that involve in a variety of physiological processes [9], and they act in concert in tumor invasion and metastasis [10]. Over the years, they have been investigated for their roles in cancer progression and metastasis [9, 11–14]. MMP14 plays an important role in CRC progression and prognosis [15]. Immunohistochemical score based on major members of MMP/TIMP profile can identify a distinct group of colorectal cancers with poor prognosis [14]. However, some MMPs, such as MMP19, have not been fully investigated in CRC. MMP19 was first isolated as an autoantigen from the synovium of a rheumatoid arthritis patient [16, 17]. MMP19 contains classical MMP structural domains, including a signal peptide, pro-peptide, catalytic domain, hinge region, and C-terminal domain [17, 18]. MMP19 is reportedly involved in the progression and metastases of various cancers, but its role in CRC remains unknown.

In this study, we used The Cancer Genome Atlas (TCGA) and whole-genome expression microarray (Gene Expression Omnibus, accession number GSE39582) databases to investigate the expression of MMP19 and RNA sequence. Furthermore, we explored the relationship between MMP19 expression and cancer prognosis using our data to determine whether MMP19 can serve as a valuable prognostic predictor in CRC patients.

## Methods

### TCGA and GSE39582 database

MMP19 mRNA expression was retrieved from the TCGA portal (http://tcga-data.nci.nih.gov) and GSE39582 database (https://www.ncbi.nlm.nih.gov/geo/). We selected patients who had both RNA sequencing data and clinicopathological factors available for the correlation analysis. The inclusion criteria were as follows: pathologically diagnosed with invasive adenocarcinoma, available intact survival information, and RNA sequencing data available. A total of 359 CRC samples in the TCGA cohort and 474 cases in the GSE39582 database were selected. The relationship between MMP19 expression and the prognosis of CRC patients was explored.

### Validation cohort

The study was approved by the Ethics Committee of Taizhou Municipal Hospital, Medical College of Taizhou University (Zhejiang, China). Before surgery, all patients provided written informed consents in compliance with the ethics of the World Medical Association (Declaration of Helsinki) for the donation of their tissue for the present research. All patients underwent radical colectomy, and all fresh tissues, including tumor tissues and normal controls, were frozen in liquid nitrogen immediately after resection and stored in RNA later at − 20 °C. Pathological diagnoses were made by at two pathologists and restaged according to the 8th American Joint Committee on Cancer guidelines. Normal control tissue was retrieved at least 10 cm from the tumor margin.

All patients were underwent followed up strategy according to NCCN guidelines. The primary endpoint for patients were OS and DFS. The OS was defined as the time from diagnoses to death from any cause, and the DFS was defined as the time from diagnoses to the first recurrence or death [19]. The survival data was got from the medical records or contacts with patients by phone or email.

### Ethics statement

This study was approved by the Taizhou Municipal Hospital Research Ethics Committee (ID: 2018-03-0039). The study was implement according to the approved guidelines. Informed consent was obtained from each patient before surgery.

### Reverse transcription-polymerase chain reaction

Reverse transcription-polymerase chain reaction (RT-PCR) was used to test the transcriptional expression levels of target genes as previously described [20, 21]. The primers were as follows: MMP19-F, 5′- GCTTCCTACTCCCCATGACAG -3′, MMP19-R, 5′- CCCATATTGTGACAGGTAGTCCA -3′. GAPDH-F, 5′-GCACCGTCAAGGCTGAGAAC-3′, GAPDH-R, 5′-TGGTGAAGACGCCAGTGGA-3′; All studies were replicated in three times.

### Immunohistochemistry (IHC) study

Immunohistochemistry (IHC) was performed on formalin-fixed, paraffin-embedded tissue sections as previous described [15, 22]. MMP19 was detected using the rabbit anti-MMP19 polyclonal antibody AP6202a (Abgent Inc.). Primary antibodies was omitted as negative control. Data were assessed by two independent single-blinded pathologists. A semi quantitative immunoreactivity scoring system was used to sort patients into high and low expression groups according to the immunoreactivity score [22, 23].

### Statistical analysis

OS, DFS or recurrence free survival (RFS) was used as primary endpoint for TCGA, GSE39582 and validation cohort. Survival analysis were compared using the univariate and multivariate Cox proportional hazard model among different MMP19 mRNA expression levels in the TCGA and GSE39582 database. The results were demonstrated as hazard ratios (HR) and 95% confidence intervals (CI). MMP19 was also classified into high and low expression subgroups in TCGA and GSE39582 cohorts by the *X*-tile program with a maximum **χ**^**2**^ value and minimum *P* value. [24]. A one-sided *P* value < 0.05 was considered as statistically significance.

## Results

### MMP19 was an independent prognostic factor for survival in the TCGA cohort

A total of 359 eligible patients were included in this study from the TCGA database, including 199 (55.4%) men and 160 (44.6%) women. The median age was 64 (range 31–90) years. The median follow-up time was 32 (range, 0–15) months and 82 patients (22.8%) died after the last follow-up.

**Fig. 1:**
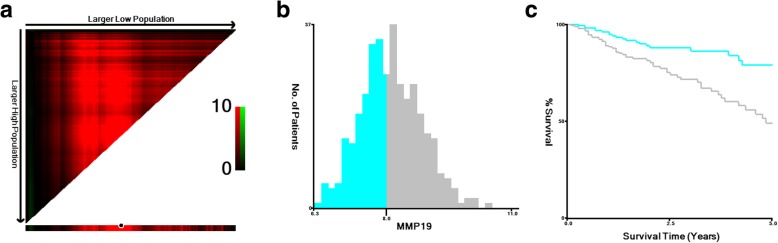
*X-*tile analyses of 5-year overall survival (OS) were performed using data from The Cancer Genome Atlas to determine the optimal cut-off values for the matrix metalloproteinase 19 (MMP19) mRNA levels. The sample of colorectal cancer (CRC) patients was equally divided into training and validation cohorts. **a** *X*-tile plots of the training sets are shown in the left panels, with plots of matched validation cohort shown in the smaller inset. **b** Optimal cut-off values highlighted by the black circles in the left panels are shown in histograms of the entire cohort (middle panels); 167 patients in the MMP19 low expression group, while 192 were in the high expression group, and (**c**) Kaplan–Meier plots are displayed in the right panels, **χ**^**2**^ = 12.602,*P* < 0.001

We first treated the MMP19 mRNA expression levels as continuous variables. MMP19 was found to be a predictor of OS in univariate Cox proportion analysis (HR: 1.449, 95%CI: 1.108–1.893, *P* = 0.007). Furthermore, age (HR: 1.029, 95%CI: 1.010–1.084, *P* = 0.002), tumor (T) stage (HR: 2.680, 95% CI: 1.694–4.242, *P* < 0.001), node (N) stage (HR = 1.759, 95% CI: 1.371–2.256, *P* < 0.001), and metastasis (M) stage (HR: 1.378, 95% CI: 1.069–1.778, *P* = 0.013) were significantly associated with prognosis. Multivariate analysis further demonstrated that MMP19 was an independent predictor of OS (HR: 1.401, 95% CI: 1.036–1.894, *P* = 0.028) (Table 1). Then, X-tile program were used to divide the patients into high (192/359, 53.48%) and low (167/359, 46.52%) MMP19 expression subgroups. The 5-year OS was 79.0 and 49.1% for those in the MMP19 low and high expression groups, respectively, and the difference was statistically significant (**χ**^**2**^ = 12.602, *P* < 0.001;Fig. 1).

**Table 1:** Univariate and multivariate Cox proportional hazards analyses of MMP19 expression and overall survival for patients with colorectal cancer in the The Cancer Genome Atlas database

Factor	Univariate analysis		Multivariate analysis	
	HR (95% CI)	*P*	HR (95% CI)	*P*
Gender	0.793(0.509–1.234)	0.303		NI
Age	1.029(1.010–1.084)	**0.002**	1.037(1.017–1.056)	**< 0.001**
T category	2.680(1.694–4.242)	**< 0.001**	2.045(1.260–3.320)	**0.004**
N stage	1.759(1.371–2.256)	**< 0.001**	1.573(1.199–2.064)	**0.001**
M stage	1.378(1.069–1.778)	**0.013**	1.092(0.829–1.440)	0.532
Tumor location	0.777(0.442–1.364)	0.379		
MMP19	1.449(1.108–1.893)	**0.007**	1.401(1.036–1.894)	**0.028**

*Abbreviations*: *CI* confidence interval, *HR* hazard ratio, *MMP19* matrix metalloproteinase 19, *T* tumor, *N* node, *M* metastasis

Bold type indicates statistical significance

### MMP19 was a predictor of recurrence-free survival in the GSE39582 cohort

To further explore the prognostic role of MMP19 in CRC, MMP19 mRNA expression was examined in another publicly available database, the GSE39582 database. The cohorts included 275(55.4%) men and 221(44.6%) women. During the follow-up period, 139 patients (28.0%) experienced tumor relapse. Univariate survival analysis demonstrated that MMP19 status, T stage, and N stage were independent factors associated with recurrence-free survival (RFS) (all *P* < 0.05). Then, the factors that were significant in univariate analyses were forward into the multivariate analysis, and the results confirmed that T stage (HR: 1.774, 95% CI: 1.274–2.468, *P* = 0.001), N stage (HR: 1.669, 95% CI: 1.343–2.073, *P* < 0.001), and MMP19 (HR: 1.470, 95% CI: 1.025–2.215, *P = 0.032*) were independent predictors for RFS (Table 2).

**Table 2:** Univariate and multivariate Cox proportional hazards analyses of MMP19 expression and relapse free survival for patients with colon cancer in the GSE39582 database

Factor	Univariate analysis		Multivariate analysis	
	HR (95% CI)	*P*	HR (95% CI)	*P*
Gender	0.744(0.528–1.048)	0.090		
Age	1.008(0.995–1.021)	0.233		
T category	1.916(1.381–2.659)	**< 0.001**	1.774(1.274–2.468)	**0.001**
N stage	1.795(1.451–2.222)	**< 0.001**	1.669(1.343–2.073)	**< 0.001**
Tumor location	1.144(0.810–1.619)	0.446		
MMP19	2.061(1.454–2.921)	**< 0.001**	1.470(1.025–2.215)	**0.032**

*Abbreviations*: *CI* confidence interval, *HR* hazard ratio, *MMP19* matrix metalloproteinase 19, *T* tumor, *N* node, *M* metastasis

Bold type indicates statistical significance

**Fig. 2:**
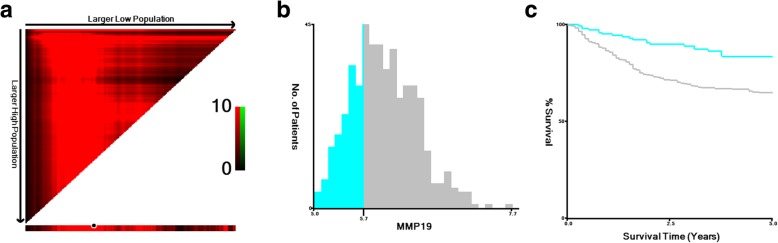
*X-*tile analyses of 5-year relapse-free survival were performed using data from the GSE39582 cohort to determine the optimal cut-off values for the MMP19 mRNA levels. **a** *X*-tile plots of the training cohort are shown in the left panels, with plots of the matched validation cohort shown in the smaller inset. **b** The optimal cut-off values highlighted by the black circles in the left panels are shown in histograms of the entire cohort (middle panels); 145 patients were in the MMP19 low expression group, while 351 were in the high expression group, and (**c**) Kaplan–Meier plots are displayed in right panels, **χ**^**2**^ = 14.070,*P* < 0.001

The X-tile program was used to divide patients into high (351/496, 70.77%) and low (145/496, 29.23%) subgroups, and the Kaplan Meier survival curve demonstrated that MMP19 overexpression was correlated with a significant decrease in RFS (**χ**^**2**^ = 12.602, *P* < 0.001; Fig. 2).

### MMP19 was correlated with inferior clinical characteristics in the validation cohort

**Fig. 3:**
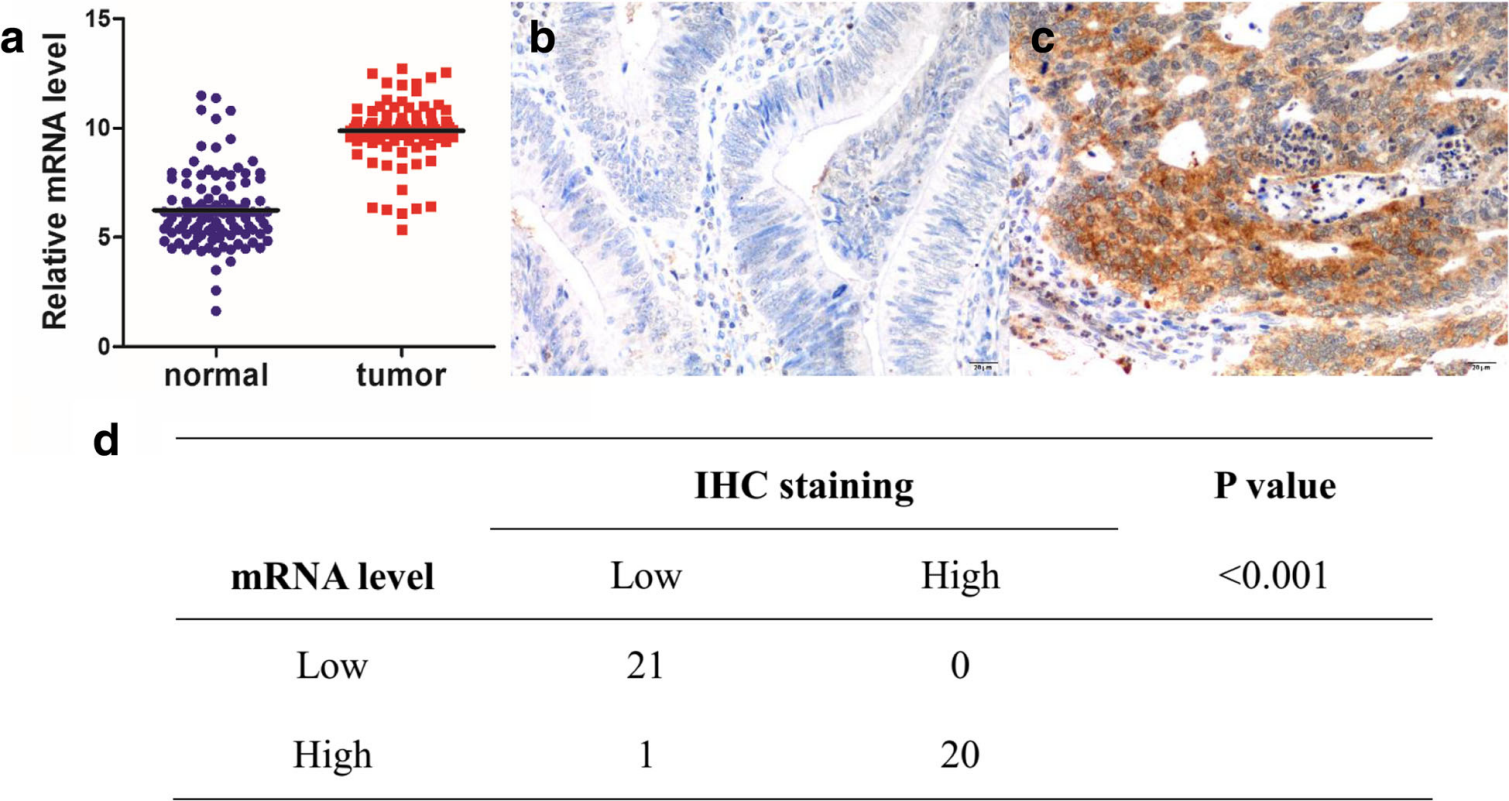
MMP19 expression pattern in CRC. **a** MMP19 expression was significantly higher in cancer tissues than in the normal controls in 198 paired tissues (*P* < 0.001).**b**, **c** Representative images of low(**b**) and high(**c**) MMP19 expression in CRC determined by IHC staining. **d** MMP19 mRNA expression is highly correlated with their protein levels (*P* < 0.001)

To investigate the potential relevance of MMP19 expression in CRC tissues in terms of clinical characteristics, MMP19 mRNA expression was further examined in 198 CRC cancer tissues and paired normal controls. The results indicated that MMP19 expression was significantly up-regulated in cancer tissues than in normal controls (*P* < 0.05; Fig. 3a). The clinical and histopathologic characteristics classified by the median MMP19 mRNA expression level are summarized in Table 3. High MMP19 expression was significantly correlated with lymph node metastases (*P* = 0.029), intramural vascular invasion (*P* = 0.015) and serum carcinoembryonic antigen status (*P* = 0.045; Table 3).

**Table 3:** Association between MMP19 expression and clinicopathological factors in colorectal cancer

Characteristics	Total	MMP19 expression		*P* value
		Low expression	High expression	
Gender				0.144
Male	122	56(56.6)	66(66.7)	
Female	76	43(43.4)	33(33.3)	
Age				0.190
≥ 60	121	65(65.7)	56(56.6)	
<60	77	34(34.3)	43(43.4)	
Primary site				0.776
colon	92	45(45.5)	47(47.5)	
rectum	106	54(54.5)	52(52.5)	
Histologic type				0.756
Adenocarcinoma	187	94(94.9)	93(93.9)	
Mucinous adenocarcinoma	11	5(5.1)	6(6.1)	
Histologic grade				0.177
Well	54	24(24.2)	30(30.3)	
Moderate	99	56(56.6)	43(43.4)	
Poor	45	19(19.2)	26(26.3)	
T stage				0.052
T2	17	10(10.1)	7(7.1)	
T3	72	43(43.4)	29(29.3)	
T4	109	46(46.5)	63(63.6)	
Node status				**0.029**
Negative	79	47(47.5)	32(32.3)	
Positive	119	52(52.5)	67(67.7)	
Intramural vascular invasion				**0.015**
Negative	156	85(85.9)	71(71.7)	
Positive	42	14(14.1)	28(28.3)	
Perineural invasion				0.692
Negative	168	85(85.9)	83(83.8)	
Positive	30	14(14.1)	16(16.2)	
CEA status				**0.045**
normal	137	75(75.8)	62(62.6)	
high	61	24(24.2)	37(37.4)	

*Abbreviations*: *CEA* carcinoembryonic antigen, *MMP19* matrix metalloproteinase 19, *T* tumor, *N* node, *M* metastasis

Bold type indicates statistical significance

Genes usually exert function through their encoded proteins. Therefore, we used immunohistochemistry to detect the expression of MMP19 protein in 42 patients in the validation group, and found MMP19 mRNA expression is highly correlated with their protein levels (*P* < 0.001) (Table 3).

### Increased MMP19 expression indicates a poor prognosis in the validation cohort

**Fig. 4:**
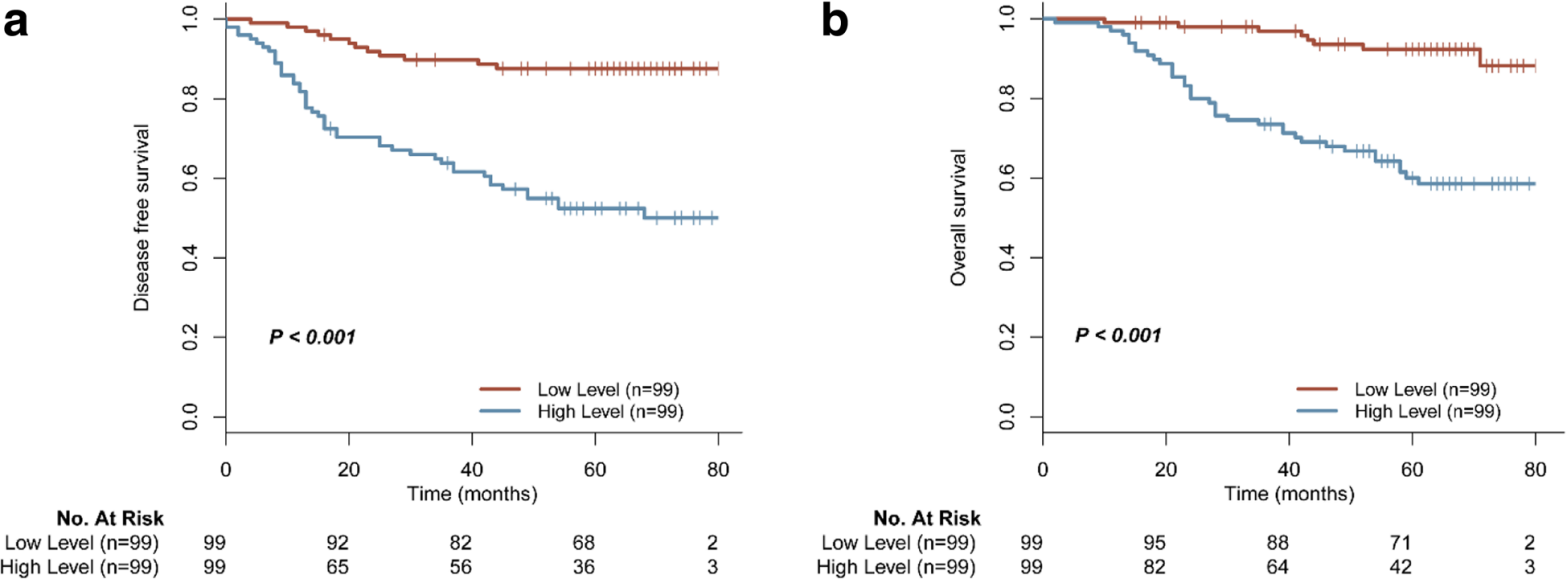
Survival analysis of CRC in the validation cohort according to MMP19 expression levels. High MMP19 expression indicates shorter (**a**) disease free survival (**χ**^**2**^ = 29.516, *P* < 0.001) and (**b**) OS (**χ**^**2**^ = 26.103, *P* < 0.001)

During a median follow-up of 54 (range, 3–89) months, 58 patients (29.3%) suffered metastasis or local recurrence, and 45 patients (22.7%) died from disease progression. The clinical significance of MMP19 in the Kaplan Meier survival curve and multivariate prognostic analysis for this cohort were in agreement. The 5-year DFS for the high and low MMP19 expression was 52.5 and 87.4%, respectively (*P* < 0.001; Fig. 4a). In the multivariate Cox regression analysis, N stage (HR: 1.642; 95%CI: 1.140–2.365; *P* = 0.008) and MMP19 expression (HR: 4.699; 95% CI: 2.461–8.974; *P* < 0.001) were independent prognostic factors for DFS (Table 4). Subjects with MMP19 expression levels above the median had shorter OS than subjects with low MMP19 levels (5-year OS: 90.3% vs. 60.0%, *P* < 0.001; Fig. 4b). In multivariate Cox regression analysis, T stage (HR: 1.854; 95%CI: 1.054–3.260; *P* = 0.032), N stage (HR: 1.856; 95%CI: 1.226–2.810; *P* = 0.003), and MMP19 expression (HR: 5.595; 95% CI: 2.573–12.164; *P* < 0.001) were independent predictor for OS (Table 5).

**Table 4:** Univariate and multivariate Cox proportional hazards analyses of MMP19 expression and disease free survival for patients with colorectal cancer in the validation cohort

Factor	Univariate analysis		Multivariate analysis	
	HR (95% CI)	*P*	HR (95% CI)	*P*
Gender	0.924(0.541–1.578)	0.772		
Age	1.327(0.790–2.227)	0.285		
T category	2.044(1.263–3.307)	**0.004**	1.527(0.972–2.400)	0.066
N stage	1.784 (1.285–2.457)	**0.001**	1.642(1.140–2.365)	**0.008**
Tumor location	0.902(0.539–1.509)	0.694		
Histologic type	0.900(0.282–2.878)	0.860		
Grade	1.200(0.831-1.733)	0.330		
Intramural vascular invasion	2.190(1.264–3.796)	**0.005**	1.337(0.754–2.371)	0.321
Perineural invasion	1.965(1.076–3.588)	**0.028**	1.393(0.738–2.628)	0.306
MMP19	4.909(2.598–9.275)	**< 0.001**	4.699(2.461–8.974)	**< 0.001**

*Abbreviations*: *CI* confidence interval, *HR* hazard ratio, *MMP19* matrix metalloproteinase 19, *T* tumor, *N* node

Bold type indicates statistical significance

**Table 5:** Univariate and multivariate Cox proportional hazards analyses of MMP19 expression and overall survival for patients with colorectal cancer in the validation cohort

Factor	Univariate analysis		Multivariate analysis	
	HR (95% CI)	*P*	HR (95% CI)	*P*
Gender	1.000(0.547–1.827)	0.999		
Age	1.416(0.788–2.543)	0.245		
T category	2.542(1.396–4.628)	**0.002**	1.854(1.054–3.260)	**0.032**
N stage	1.975 (1.354–2.881)	**< 0.001**	1.856(1.226–2.810)	**0.003**
Tumor location	0.808(0.450–1.450)	0.475		
Histologic type	1.206(0.374–3.893)	0.754		
Grade	1.155(0.762-1.751)	0.496		
Intramural vascular invasion	2.419(1.312–4.458)	**0.005**	0.927(0.435–1.975)	0.210
Perineural invasion	1.412(0.680–2.932)	0.355		
MMP19	5.807(2.702–12.483)	**< 0.001**	5.595(2.573–12.164)	**< 0.001**

*Abbreviations*: *CI* confidence interval, *HR* hazard ratio, *MMP19* matrix metalloproteinase 19, *T* tumor, *N* node

Bold type indicates statistical significance

## Discussion

With recent advances in high-throughput technologies (e.g., RNA deep sequencing), the transcriptomes of many tumors have been surveyed and many novel biomarkers and therapeutic targets have been identified. To validate MMP19 as a potential novel target gene to predict survival, we designed our study in three steps. First, we found MMP19 as a potential novel biomarker in terms of survival in the TCGA database. Second, we studied MMP19 in the GSE39582 database and confirmed it as novel biomarker for CRC. Finally, because some important clinical characteristics were missed in the TCGA and GSE39582 database, such as strategies of adjuvant therapy, and the quality of surgery,, we further validated the results from our own database. We found that MMP19 expression was significantly upregulated in cancer tissues relative to the normal controls, and that high MMP19 expression was associated with inferior clinical characteristics. Importantly, MMP19 was validated as an independent predictor for both OS and DFS for CRC after colectomy. Our results point to a crucial role for MMP19 in the evolution of CRC.

MMP19 is a classical member of the MMP family, which consists of at least 23 enzymes [17]. MMP19 shares the typical structural domains of MMP, including a signal peptide, propeptide, catalytic domain, hinge region, and C-terminal domain [18]. MMP19 has been gradually recognized as an important oncogene in carcinogenesis and progression. It is associated with increased mortality and promotes metastatic behavior in non-small cell lung cancer (NSCLC) [25]. An increase in MMP19 expression indicates the progression of cutaneous melanoma and might augment melanoma growth by promoting the invasion of tumor cells [26]. MMP19 is highly expressed in astroglial tumors and promotes the invasion of glioma cells [27]. For CRC, a previous study reported that MMP19 may involve in malignant transformation, is low expressed in normal mucosa, and is upregulate during neoplastic progression [28], but its prognostic value has not been reported. Here, we provide new evidence that MMP19 plays a crucial role in CRC. High MMP19 expression was significantly correlated with lymph node metastases and intramural vascular invasion, which suggests that MMP19 may play a critical role in CRC invasion and metastases. Distant metastases and recurrence are two main reasons for cancer-related death; thus, it was not surprising that high MMP19 expression was correlated with a poor prognosis in CRC. Similar, increased MMP19 gene expression correlates with a worse prognosis and facilitates invasion in NSCLC [25].

Although we obtained conclusions from three independent databases, there were some limitations in our study. First, we only investigated the clinical significance of MMP19 in CRC using patient samples; no in vitro study or animal models were used in this study. Second, a further study is needed to understand the mechanisms underlying the function of MMP19 in CRC progression.

## Conclusion

In summary, our results demonstrate that MMP19 is upregulated in CRC and is a potential predictor of CRC, which provides additional information for predicting survival and developing a therapeutic strategy. Our results warrant further studies on the detailed mechanisms by which MMP19 facilitates tumor progression in CRC.

## Abbreviations

CRC: Colorectal cancer
GEO: Whole-genome expression microarray
MMP19: Matrix metalloproteinases 19
MMR: Mismatch repair protein
OS: Overall survival
RFS: Recurrence free survival
TCGA: The Cancer Genome Atlas
